# Highly active antiretroviral therapy and dyslipidemia in people living with HIV/AIDS in Fako Division, South West Region of Cameroon

**DOI:** 10.1186/s12872-015-0090-5

**Published:** 2015-08-28

**Authors:** Dickson Shey Nsagha, Elroy Patrick Weledji, Nguedia Jules Clement Assob, Longdoh Anna Njunda, Elvis Asangbeng Tanue, Odette Dzemo kibu, Charlotte Wenze Ayima, Marcelin Ngowe Ngowe

**Affiliations:** Department of Public Health and Hygiene, Faculty of Health Sciences, University of Buea, P.O Box 12, Buea, Cameroon; Department of Surgery, Obstetrics and Gynaecology, Faculty of Health Sciences, University of Buea, P.O Box 12, Buea, Cameroon; Department of Medical Laboratory Sciences, Faculty of Health Sciences, University of Buea, P.O Box 12, Buea, Cameroon

## Abstract

**Background:**

The advent of HAART has been associated with a profound reduction in morbidity and mortality from HIV/AIDS. However, side effects and toxicities associated with HAART may lead to an increased risk for cardiovascular diseases. The aim of this study was to determine the prevalence of dyslipidemia and determining factors of derangements in lipid profile associated with the use of HAART regimens in people living with HIV/AIDS in Fako Division of the South West Region of Cameroon.

**Methods:**

This cross-sectional study was conducted between March and August 2014. Lipid profile was determined after overnight fast and dyslipidemia diagnosed according to the US National Cholesterol Education Program III criteria. Socio-demographic characteristics were also collected using a questionnaire. Data was analyzed using STATA; chi-square test, student’s *t*-test, ANOVA and logistic regressions were computed.

**Results:**

Two hundred and nine participants were recruited including 157 (75.1 %) on HAART and 52 (24.9 %) HAART-naïve. Antiretrovirals were drugs containing two nucleoside backbones (zidovudine/ /lamivudine/tenofovir) with either a non-nucleoside (nevirapine/efavirenz) or a protease inhibitor (lopinavir). No patient was treated with statins. Their mean age was 43.4 (±11.0) years. The mean CD4^+^ T cell count was 425 (±281) cells/μl after mean duration of HIV infection of 54.8 (±43.9) months and mean duration on ART of 63.7 (±41.4) months. The prevalence of total cholesterol (≥ 200 mg/dL) was 51.0 % in patients on HAART and 9.6 % pre-HAART patients (*p* < 0.0001), whereas LDL-cholesterol ≥ 130 mg/dL occurred in 36.9 % and in 7.7 % respectively, (*p* = 0.0001). Receiving HAART (adjusted odds ratio =6.24, 95 % CI: 2.33–17.45, *p* < 0.0001) and HIV duration of 42 months and more (aOR = 2.26, 95 % CI: 1.16–4.42, *p* = 0.017) were independently associated with total cholesterol ≥ 200 mg/dL. Receiving HAART (aOR = 5.28, 95 % CI: 1.17–16.32, *p* = 0.004) was independently associated with raised LDL-cholesterol values. The adjusted odds ratio (95 % CI) of BMI ≥ 25.0 kg/m^2^ versus BMI < 25.0 kg/m^2^ was 3.25 (1.44–7.34) for triglycerides ≥ 150 mg/dL.

**Conclusion:**

HAART regimens were significantly associated with atherogenic lipid profile. Lipid profile should be monitored in HIV/AIDS patients on therapy so that any negative effects of HAART are optimally managed.

## Background

The introduction and widespread use of combination antiretroviral therapy (ART) referred to as highly active antiretroviral therapy (HAART) in the mid 1990’s, has led HIV-infected individuals to experience a dramatic decline in immunodeficiency-related events, including causes of death [1]. The advent of HAART has been associated with a profound reduction in morbidity and mortality from HIV/AIDS [2]. However, antiretroviral drugs also have side effects of varying severity. Disorders of lipid metabolism associated with HAART have been largely described in the developed and developing countries, mainly in patients on treatment regimens including protease inhibitors (PIs) and stavudine [3]. Treatment regimens including nevirapine and efavirenz have also been reported to induce lipid derangements [4]. ART can induce raised levels of total cholesterol (TC), LDL-cholesterol (LDL-c) and triglycerides (TG), and variables effects on HDL-cholesterol (HDL-c) levels [3]. Also, several reports have documented increased prevalence of hypertriglyceridemia, and low HDL-c in both HAART-treated and HAART-naïve patients [5]. Side effects and toxicities are associated with these highly effective therapies and there is growing concern that the metabolic complications associated with HIV and antiretroviral therapy may lead to an increased risk for cardiovascular diseases [6]. These ART-induced lipid derangements are potentially atherogenic and can increase cardiovascular risk [7]. There is scarcity of information in support of lipid profile derangements associated with HAART use in sub-Saharan Africa [8]. The aim of the present study was to determine the prevalence of dyslipidemia and determining factors of derangements in lipid profile associated with the use of HAART regimens in people living with HIV/AIDS in Fako Division of the South West Region of Cameroon.

## Methods

### Study setting and participants

This was a hospital-based cross-sectional study. Participants were recruited between March and August 2014 at treatment centers of people living with HIV/AIDS (PLWHA) in the Limbe and Buea Regional Hospitals in Fako Division, South West Region of Cameroon. Two groups of participants were selected. Group one was made up of individuals living with HIV/AIDS who had been receiving HAART for at least 6 months and reported by the treatment centres as non-defaulters (ART group). The second group was made up of individuals newly diagnosed with HIV but who were not yet receiving HAART (ART-naïve group). All participants were 21 years of age and above. Participants receiving lipid altering therapies, pregnant women, known diabetes mellitus patients, those with renal failures and patients who refused to be part of the study were excluded.

### Data collection and measurements

For all participants, structured questionnaires were used to collect data on the socio-demographic characteristics and patients’ record were reviewed for information on their current CD4^+^ T cell count, HIV and HAART status. Body mass index and blood pressure were also measured. About 5 ml of venous blood sample was collected from each participant through venipuncture after a 12 h overnight fast and centrifuged at 2500 cycles/min for 10 min, and serum was obtained for fasting blood sugar and lipid profiles measurements. Fasting blood sugar and lipid profile were assessed through enzymatic methods (INMESCO, GmbH (L-S 04/2009) for all participants including total cholesterol (TC), high density lipoprotein cholesterol (HDL-c), low density lipoprotein-cholesterol (LDL-c) and triglycerides (TG). The TC/HDL-c ratio was also calculated. In accordance with the US National Cholesterol Education Program, Adult Treatment Panel III (NCEP-ATP III) guidelines, abnormal lipid profile was defined as TC ≥ 200 mg/dL, HDL-c < 40 mg/dL, LDL-c ≥ 130 mg/dL, TG ≥ 150 mg/dL and TC/HDL-c ratio ≥ 5 [9].

### Ethical considerations

Ethical Clearance was obtained from the Institutional Review Board of the Faculty of Health Sciences of the University of Buea (Reference N^o^ 2014–02–0193). Administrative authorization was obtained from the Regional Delegation of the Ministry of Public Health and the District Health Services. The purpose of the study and the role of the participants were well explained in the consent form to the participants and participation could only take place after the participant had read and signed the informed consent form voluntarily.

### Data analysis

Data entry and Database management was computed using Microsoft excel 2010 (Microsoft Corporation Inc, USA). Statistical analyses were done using STATA version 10.1. Chi-square test was used to evaluate differences in frequency distribution. Student’s *t*-test and analysis of variance (ANOVA) were used to assess differences between group means. A single model logistic regression was computed to determine the association of independent factors with abnormal level of each lipid profile. *P*-value less than 0.05 were considered as statistically significant at 95 % confidence level.

## Results

### Demographic characteristics of participants

A total number of 209 participants were enrolled in the study, with 51 (24.4 %) males and 158 (75.6 %) females. Two groups of patients were investigated: the first group of 157 HIV-infected patients [34 (21.7 %) males, 123 (78.3 %) females], were currently receiving HAART; the second group of 52 HIV-infected patients [17 (32.7 %) males, 35 (67.3 %) females], were HAART inexperienced. The mean age of individuals who were on HAART was 44.9 (±10.7) years while that of pre-HAART was 38.8 (±10.8) years (Table 1). The distribution of first-line ART regimens were as follows: AZT/3TC/NVP (80 participants), AZT/3TC/EFV (2 participants), TDF/3TC/NVP (21 participants) and TDF/3TC/EFV (41 participants). The second-line ART regimen was TDF/3TC/LPVr with 13 participants. Among HAART patients, 82 (52.2 %) were on zidovudine (AZT), 75 (47.8 %) on tenofovir (TDF), 101 (64.3 %) on nevirapine (NVP), 43 (27.4 %) on efavirenz (EFV) and 13 (8.3 %) on lopinavir (LPVr). All regimens included 3TC. ART patients who had been on treatment for an average of 64 (±41) months were reported as non-defaulters within the past six months. The mean CD4^+^ T cell count in the two groups was 425 (±280) cells/μl after a mean duration of HIV infection of 54.8 (±43.9) months. HAART experienced patients had been on HAART for a mean duration of 63.7 (±41.4) months. Thirty eight (18.2 %) patients reported drinking alcohol but only four admitted they were current cigarette smokers. Obesity, hypertension and diabetes mellitus were screened in 48 (23.0 %), 46 (22.0 %) and 5 (2.4 %) of the participants respectively (Table 1).

**Table 1:** General characteristics of the study population by HAART status

Variables		HAART experienced Group (*n* = 157)	HAART-naïve Group (*n* = 52)	Total	*p*-value
Gender	Male, n(%)	34 (21.7)	17 (32.7)	51 (24.4)	
	Female, n(%)	123 (78.3)	35 (67.3)	158 (75.6)	
Age (years)	Mean ± SD	44.9 (10.7)	38.8 (10.8)	43.4 (11.0)	0.0002
	21-30 years, n(%)	12 (7.6)	12 (23.1)	24 (11.5)	
	31-40 years, n(%)	48 (30.6)	19 (36.5)	67 (32.1)	
	41-50 years, n(%)	52 (33.1)	15 (28.8)	67 (32.1)	
	> 50 years, n(%)	45 (28.7)	6 (11.5)	51 (24.4)	
BMI (kg/m^2^)	Mean ± SD	27.0 (5.4)	25.3 (4.6)	26.6 (5.3)	0.0188
	< 18 kg/m^2^, n(%)	3 (1.9)	1 (1.9)	4 (1.9)	
	18-24.9 kg/m^2^, n(%)	64 (40.8)	2548.1)	89 (42.6)	
	25.0-29.9 kg/m^2^, n(%)	50 (31.8)	18 (34.6)	68 (32.5)	
	≥ 30 kg/m^2^, n(%)	40 (25.5)	8 (15.4)	48 (23.0)	
CD4^+^ T cell count*	Mean ± SD	430.3 (301.2)	411 (222.5)	424.9 (280.6)	0.7389
	< 200 cells\μl	20 (23.5)	6 (18.2)	26 (22.0)	
	200-499 cells\μl	36 (42.4)	14 (42.4)	50 (42.4)	
	≥ 500 cells\μl	29 (34.1)	13 (39.4)	42 (35.6)	
Alcohol intake	No, n(%)	131 (83.4)	40 (76.9)	171 (81.8)	
	Yes, n(%)	26 (16.6)	12 (23.1)	38 (18.2)	
Cigarette smoking	No, n(%)	154 (98.1)	51 (98.1)	205 (98.1)	
	Yes, n(%)	03 (1.9)	01 (1.9)	04 (1.9)	
Hypertension	Not present, n(%)	118 (75.2)	45 (86.5)	163 (78.0)	
	Present, n(%)	39 (24.8)	07 (13.5)	46 (22.0)	
Diabetes Mellitus	Not present, n(%)	154 (98.1)	50 (96.2)	204 (97.6)	
	Present, n(%)	03 (1.9)	02 (3.8)	05 (2.4)	
Obesity	Not present, n(%)	117 (74.5)	44 (84.6)	161 (77.0)	
	Present, n(%)	40 (25.5)	08 (15.2)	48 (23.0)	

*HAART*-Highly Active Antiretroviral Therapy, *CD*-Cluster of differentiation, *SD*-Standard Deviation, *-CD4+ T cell count values available for *n* = 118

### Dyslipidemia and characteristics of lipid profiles

The mean TC and mean LDL-c were significantly higher in patients on HAART than in HAART-naïve patients (*p* < 0.001). The mean TG and TC/HDL-c were significantly higher in participants on HAART than their HAART inexperienced counterparts (*p* < 0.05). There was no statistically significant difference between the two groups for mean HDL-c (Table 2). The prevalence of TC ≥ 200 mg/dL and LDL-c ≥ 130 mg/dL were significantly higher in the HAART treated group when compared to the pre-HAART group. However, the prevalence of HDL-c below 40 mg/dL, TG ≥ 150 mg/dL and TC/HDL-c ≥ 5 were not statistically significantly different in pre-HAART group when compared to those on HAART treatment (Table 2).

**Table 2:** Serum lipid profiles of study participants by HAART status

Parameters	HAART experienced Group (*n* = 157)	HAART naïve Group (*n* = 52)	*p*-value
Total cholesterol, mean ± SD	208.7 (71.9)	147.4 (44.6)	< 0.0001
≥ 200 mg/dL	80 (51.0 %)	5 (9.6 %)	< 0.0001
LDL-cholesterol, mean ± SD	117.6 (68.2)	65.9 (42.0)	< 0.0001
≥ 130 mg/dL	58 (36.9 %)	4 (7.7 %)	0.0001
HDL-cholesterol, mean ± SD	67.0 (62.6)	62.5 (26.3)	0.6153
< 40 mg/dL	22 (14.0 %)	7 (13.5 %)	0.9257
Triglycerides, mean ± SD	117.2 (59.3)	97.7 (57.5)	0.0396
≥ 150 mg/dL	32 (20.4 %)	9 (17.3 %)	0.6257
TC/HDL-cholesterol ratio, mean ± SD	3.7 (2.6)	2.8 (1.7)	0.0205
≥ 5	24 (15.3 %)	3 (5.8 %)	0.0769

*HAART*- Highly Active Antiretroviral Therapy, TC-Total Cholesterol; *HDL*-High-Density Lipoprotein, *LDL*-Low- Density Lipoprotein, SD-Standard Deviation

### Total cholesterol and triglyceride levels differed among the HAART regimens

The mean TC was higher in patients on TDF than those on AZT among the nucleoside reverse transcriptase inhibitor (NRTIs) based treatment. Among the non-nucleoside reverse transcriptase inhibitor (NNRTIs) based treated patients, the mean TC was also higher in patients on EFV compared to NVP. Although this was not statistically significant, it was above the normal cut off point. The mean TG was statistically significantly higher in patients on TDF compared to AZT, and EFV compared to NVP (*p* = 0.009 and 0.004 respectively) (Table 3). No significant difference was observed in the prevalence of lipid profile derangements between patients receiving AZT compared to those on TDF; and patients treated with EFV compared to those treated with NVP (Table 3). No significant difference was observed in the mean lipid derangements, and the percentages between protease inhibitors (PIs) treated and non-PIs treated patients. However, the mean TG value was slightly higher in PIs based patients (146.8 ± 59.3) compared to non-PIs based (114.5 ± 59.5) (*p* = 0.0604).

**Table 3:** Prevalence of abnormal lipid profiles among patients treated with NRTIs and NNRTIs based antiretroviral regimens

NRTIs based regimens			
Lipid profile	AZT based (*n* = 82)	TDF based (*n* = 75)	*p*-value
Total cholesterol, mean ± SD	205.2 (66.3)	212.3 (77.8)	0.540
≥ 200 mg/dL	41 (50.0 %)	39 (52.0 %)	0.802
LDL-cholesterol, mean ± SD	115.9 (63.2)	119.5 (73.6)	0.744
≥ 130 mg/dL	27 (32.9 %)	31 (41.3 %)	0.276
HDL-cholesterol, mean ± SD	70.1 (29.9)	63.7 (25.6)	0.153
< 40 mg/dL	13 (15.9 %)	9 (12.0 %)	0.482
Triglycerides, mean ± SD	102.4 (46.9)	133.4 (67.1)	0.009
≥ 150 mg/dL	12 (14.6 %)	20 (26.7 %)	0.060
TC/HDL-cholesterol ratio, mean ± SD	3.6 (2.7)	3.9 (2.4)	0.405
≥ 5	12 (14.6 %)	12 (16.0 %)	0.808
NNRTIs based regimens			
	NVP based (*n* = 101)	EFV based (*n* = 43)	
Total cholesterol, mean ± SD	204.1 (65.5)	218.7 (90.6)	0.282
≥ 200 mg/dL	49 (48.5 %)	23 (53.5 %)	0.583
LDL-cholesterol, mean ± SD	114.6 (61.2)	126.5 (87.7)	0.353
≥ 130 mg/dL	36 (35.6 %)	17 (39.5 %)	0.657
HDL-cholesterol, mean ± SD	68.3 (29.5)	63.8 (22.5)	0.370
< 40 mg/dL	17 (16.8 %)	3 (7.0 %)	0.119
Triglycerides, mean ± SD	105.4 (29.5)	136.0 (75.4)	0.004
≥ 150 mg/dL	15 (14.9 %)	12 (27.9 %)	0.068
TC/HDL-cholesterol ratio, mean ± SD	3.6 (2.7)	3.9 (2.3)	0.635
≥ 5	16 (15.8 %)	7 (16.3 %)	0.940

*HAART*- Highly Active Antiretroviral Therapy, *TC*-Total Cholesterol, *HDL*-High-Density Lipoprotein, *LDL*-Low- Density Lipoprotein, *AZT*-Zidovudine, *TDF*-Tenofovir, *NVP*-Nevirapine, *EFV*-Efavirenz, *SD*-Standard Deviation

### Dyslipidemia with CD4^+^ T cell count, hypertension, diabetes mellitus and obesity

No significant difference was observed in the mean lipid profile parameters in the different CD4^+^ T cell count categories. HAART experienced participants with CD4+ T cell counts < 200 cells/μl and ≥ 500 cells/μl had higher mean TC values (227.9 (±70.7) and 230.0 (±101.7) respectively. No significant difference was found between mean values of lipid derangements and presence or absence of hypertension and diabetes mellitus. However, hypertensive patients had higher mean values of raised TC (203.1 ± 80.6) compared to non-hypertensive (190.6 ± 68.3) (*p* = 0.2942). Obese patients had higher mean values of raised TC (218.4 ± 83.1) compared to the non-obese (185.9 ± 65.7) (*p* = 0.0052). Also, obese patients had higher mean values of LDL-c (128.5 ± 76.5) compared to the non-obese (97.7 ± 61.7) (*p* = 0.0045).

### Association of gender, BMI, HIV duration and HAART use with abnormal lipid profiles

The female gender was significantly and positively associated with raised TC (OR: 2.03, 95 % CI: 1.03-4.98, *p* = 0.042). The BMI of 25 kg/m^2^ and higher was significantly associated with raised TG (OR: 0.30, 95 % CI: 0.12-0.75, *p* = 0.010). The duration of HIV infection of 42 months and higher was significantly associated with raised TC and increased TG (*p* = 0.045 and 0.035 respectively). The odds ratio (95 % CI) of HIV infection duration of 42 months and higher versus less than 42 months was 0.51 (0.26-0.99) for TC ≥ 200 mg/dL and 0.36 (0.14-0.93) for TG ≥ 150 mg/dL. Being on HAART treatment for more than 55 months was significantly associated with raised TC and raised TG, with 95 % CI odds ratio of 0.47 (0.25-0.90) and 0.31 (0.13-0.74) respectively (Table 4).

**Table 4:** Association of variables with abnormal lipid profile levels among HAART treated participants

Explanatory variables	TC ≥ 200 mg/dL UOR (95 % CI)	LDL-c ≥ 130 mg/dL UOR (95 % CI)	HDL-c < 40 mg/dL UOR (95 % CI)	TG ≥ 150 mg/dL UOR (95 % CI)
Gender (Female)	2.3 (1.03-4.98)	1.29 (0.58-2.89)	0.54 (0.20-1.44)	0.52 (0.22-1.25)
*p*-value	0.042	0.532	0.217	0.144
Age > 40 years	0.81 (0.43-1.56)	1.18 (0.61-2.31)	0.93 (0.36-2.37)	0.70 (0.30-1.60)
*p*-value	0.535	0.621	0.879	0.391
BMI ≥ 25 kg/m2	0.80 (0.42-1.51)	0.82 (0.43-1.59)	0.92 (0.37-2.30)	0.30 (0.12-0.75)
p-value	0.490	0.558	0.857	0.010
CD4^+^ T cell count < 200 cells/μl	0.80 (0.29-2.17)	0.72 (0.26-1.97)	0.58 (0.13-2.55)	0.64 (0.17-2.36)
*p*-value	0.652	0.518	0.468	0.506
HIV duration ≥ 42 months	0.51 (0.26-0.99)	0.59 (0.29-1.18)	0.66 (0.24-1.79)	0.36 (0.14-0.93)
p-value	0.045	0.136	0.413	0.035
HAART duration > 55 months	0.47 (0.25-0.90)	0.58 (0.30-1.11)	0.77 (0.31-1.92)	0.31 (0.13-0.74)
*p*-value	0.022	0.101	0.572	0.008
Hypertension	1.02 (0.49-2.10)	0.94 (0.44-2.00)	0.87 (0.30-2.54)	0.81 (0.32-2.06)
*p*-value	0.962	0.876	0.805	0.664
Diabetes Mellitus	0.47 (0.04-5.34)	0.85 (0.08-9.59)	NA	8.27 (0.73-94.2)
*p*-value	0.546	0.896	NA	0.089
Obesity	1.88 (0.90-3.92)	2.08 (1.00-4.32)	1.11 (0.40-3.08)	1.44 (0.61-3.38)
*p*-value	0.093	0.050	0.835	0.403
Alcohol intake	1.38 (0.59-3.24)	0.72 (0.29-1.78)	1.14 (0.35-3.70)	2.49 (0.99-6.27)
*p*-value	0.453	0.476	0.825	0.054

*TC*-Total Cholesterol, *LDL*-c-Low-Density Lipoprotein Cholesterol, *HDL-c*-High-Density Lipoprotein Cholesterol, *TG*-Triglyceride, *HAART*-Highly Active Antiretroviral Therapy, *UOR*-Unadjusted Odds Ratio, *BMI*-Body Mass Index, *CD*-Cluster of differentiation, *NA*- not applicable; Reference category: Male, Age ≤ 40 years, BMI < 25 kg/m^2^, CD4^+^ T cell count ≥ 200 cells/μl, HIV duration < 42 months, HAART duration ≤ 55 months, non-hypertensive, non-diabetic, non-obese, no alcohol intake

Adjusting for potential confounding factors such as gender, age, BMI, duration with HIV and HAART initiation, receiving HAART was significantly and positively associated with raised TC and LDL-c (*p* < 0.0001 and 0.004 respectively). The adjusted odds ratio (95 % CI) of HAART-treated versus HAART-naïve was 6.24 (2.33–17.45) for TC ≥ 200 mg/dL and 5.28 (1.17–16.32) for LDL-c 130 mg/dL. Antiretroviral therapy was not positively associated with decreased HDL-c, increased TG and raised TC/HDL-c ratio (*p* = 0.904, 0.762 and 0.188 respectively). Raised body mass index (BMI ≥ 25.0 kg/m^2^) was significantly and positively associated with increased TG (aOR: 3.25, 95 % CI: 1.44-7.34, *p* = 0.005). The duration of HIV infection of 42 months and higher was significantly and positively associated with raised TC (aOR: 2.26, 95%CI: 1.16-4.42, *p* = 0.017). HAART treatment was positively associated with the ratio of TC/HDL-c but this was not significant (Table 5).

**Table 5:** Association of HAART and other variables with dyslipidemia among HIV-infected patients

Explanatory Variables	TC ≥ 200 mg/dL AOR (95 % CI)	LDL-c ≥ 130 mg/dL AOR (95 % CI)	HDL-c < 40 mg/dL AOR (95 % CI)	TG ≥ 150 mg/dL AOR (95 % CI)	TC/HDL-c ≥ 5 AOR (95 % CI)
Antiretroviral therapy					
HAART-naïve*	1.00	1.00	1.00	1.00	1.00
HAART treated	6.24 (2.33-17.45)	5.28 (1.17-16.32)	1.07 (0.37-3.05)	0.86 (0.32-2.28)	2.51 (0.64-9.89)
*p*-value	< 0.001	0.004	0.904	0.762	0.188
Gender					
Male*	1.00	1.00	1.00	1.00	1.00
Female	2.17 (0.99-4.74)	1.20 (0.55-2.65)	0.45 (0.19-1.07)	0.44 (0.19-1.01)	0.42 (0.16-1.09)
*p*-value	0.053	0.645	0.071	0.054	0.076
Age group					
≤ 40 years*	1.00	1.00	1.00	1.00	1.00
> 40 years	1.11 (0.58-2.31)	0.91 (0.47-1.78)	0.80 (0.35-1.84)	1.22 (0.57-2.58)	1.02 (0.42-2.46)
p-value	0.746	0.789	0.605	0.611	0.963
Body Mass Index					
BMI < 25 kg/m^2^*	1.00	1.00	1.00	1.00	1.00
BMI ≥ 25 kg/m^2^	0.98 (0.52-1.86)	1.07 (0.56-2.05)	0.88 (0.39-1.98)	3.25 (1.44-7.34)	2.19 (0.87-5.51)
*p*-value	0.962	0.845	0.757	0.005	0.097
HIV duration					
< 42 months*	1.00	1.00	1.00	1.00	1.00
≥ 42 months	2.26 (1.16-4.42)	1.71 (0.85-3.45)	1.32 (0.53-3.29)	1.89 (0.81-4.41)	1.58 (0.60-4.13)
*p*-value	0.017	0.133	0.0.545	0.140	0.350

*TC*-Total Cholesterol, *LDL-c*-Low-Density Lipoprotein Cholesterol, *HDL-c*-High-Density Lipoprotein Cholesterol, *TG*-Triglyceride; *HAART*-Highly Active Antiretroviral Therapy, *AOR*-Adjusted Odds Ratio, *BMI*-Body Mass Index; *-Reference category

## Discussion

The aim of this study conducted in a resource-poor setting was to determine the prevalence of dyslipidemia and determining factors of derangements in lipid profile associated with the use of HAART regimens in HIV/AIDS patients. We found that CD4^+^ T cell count in the HAART group was higher but not significantly different from the HAART-naïve group. Though not significantly different, this shows the effects of HAART in improving the immunological properties of the HIV treated participants. Our finding is contrary to that observed by D’Ascenzo and colleague [10] where CD4^+^ T cell count of < 200 cells/μl was associated with increased risk of myocardial infarction and cardiovascular instability. The reason for lack of association between lipid parameters in our cohort of HIV/AIDS patients and the immune status may be related to the close similarity in the CD4^+^ T cell count as most patients were in the CD4^+^ T cell count range 200–499 cells/μl and above. The study has demonstrated a high prevalence of lipid derangements (64.3 %) in HIV patients receiving HAART. Though high, the prevalence of dyslipidemia in our study is similar to the reported rate of 70.2 % in patients receiving ART in a rural Cameroonian population [11], but lower than that observed in an urban population of Southern Ethiopia with 82.3 % [12], and 76.0 % observed in HAART-naïve patients in Tanzania [13]. We found that the proportions of hypercholesterolemia and raised levels of LDL-c were significantly higher in the HAART group compared to the HAART-naïve group. The described raised lipid profiles (TC and LDL-c) are atherogenic [9, 14], and were still present even after adjusting for confounders and suggests a potential risk for the development of cardiovascular diseases in a significant proportion of HIV-infected patients in the near future. We found that the prevalence of raised TC in the HAART group was high. This prevalence is higher than that reported from two similar studies in Cameroon [11, 15], and that found in rural Ugandans [8], where participants were followed for 24 months for lipid derangements. More than 50 % of our HAART experienced participants were either overweight or obese and this might have a contributing factor on raised TC. Moreover, the mean TC was also significantly higher in the obese participants compared to the non-obese. However, there are suggestions that the magnitude of HAART induced lipid derangements could vary across populations and settings. The prevalence of high LDL-c in our study was lower than that reported in urban Cameroon (43.5 %) [15], but higher than that observed in rural Cameroon (33.3 %) [11], and similar to the prevalence reported from India [16]. However, the mean LDL-c was significantly higher in the obese participants compared to the non-obese. The prevalence of raised TG in our study was lower than that reported in an urban Cameroon study (46.4 %) [15], and in the rural Cameroon setting (51.8 %) [11], and India [16], but similar to that reported in Kenya [17]. We found comparable percentages of HAART experienced group (14.0 %) and pre-HAART (13.5 %) who had decreased HDL-c. The HDL-c level was unaffected by HAART status in our study which is not in accordance with the findings of Pujari and colleagues [18] in whose study an 18-month treatment with first-line ART regimens was associated with significant increase in HDL-c level.

Several studies have found that stavudine was more involved in the occurrence of lipid derangements as compared with other NRTIs [19–22]. However, instead of stavudine, our participants were either on tenofovir or zidovudine. We found no difference in lipid profiles (TC, LDL-c and HDL-c) when participants on tenofovir were compared to those on zidovudine. This is in line with the findings of Buchacz and colleagues in Uganda [8], and those of Yone and colleagues in Cameroon [15]. We observed significantly higher TG in patients on tenofovir compared to those on zidovudine. Most of the patients on tenofovir were also on lopinavir, a protease inhibitor that has been associated with adverse lipid profiles [23, 24]. In line with the reports from rural Uganda [8], Cameroon [15] and India [4], we found no significant difference in lipid profiles (TC, LDL-c and HDL-c) of patients on efavirenz compared to those on nevirapine. In the present study, the raised TC and LDL-c were significantly and positively associated with the use of HAART treatment, and the findings are in line with another study conducted in Cameroon [15]. Moreover, NNRTIs have been reported to derange lipid profiles during therapy [25]. However, supportive evidences are very scarce in Sub-Sahara African countries concerning lipid derangements in patients receiving NNRTIs treatment regimens [8, 26]. We found a similar scenario with other cross-sectional studies [11, 15, 27] carried out in resources-constrained settings with reported prevalence of high level of total cholesterol ranging from 23 to 41 % in patients treated with NNRTI based regimens [27].

The association between HAART and adverse lipid profile has been largely described for regimens that include PIs [23, 24], but this is contrary to our findings. This may be due to the small number of patients treated with PIs in our study. In this study, high TC and LDL-c were associated with HAART use. High TC was also associated with age >40 years, but was no association could be found after adjusting for confounding factors. The association of high TC with age is well known as its risk increases with increased age [13, 28].

There is lack of data on HIV viral load to clearly define disease stages in the two groups. However, the CD^+^ T cell counts gave a snapshot of disease progression in the untreated group and disease suppression in the treated group. Comprehensive cardiovascular risk stratifications were not assessed in this study. However, the increased risk of atherothrombotic cardiovascular disease associated with the described lipid derangement is well known [8, 29] and the long term use of HAART may have an impact on cardiovascular health. Our study, by nature is cross-sectional and inference about causal relationship is not possible. Cohort studies could monitor lipid profile alterations during HAART, and their potential impact on cardiovascular health of people living with HIV. Subsequent studies could address the issue of the small number of male participants, small number of patients in the HAART untreated group, and the lack of HIV-negative controls that our study could not handle.

## Conclusion

This study indicates that HIV-infected patients receiving WHO-recommended HAART treatment have a high prevalence of lipid profile derangements when compared to the HAART non-treated HIV-infected patients. Uses of HAART regimens are significantly associated with atherogenic lipid profiles. Lipid profile and other cardiovascular risk factors should be monitored in patients on ART so that any negative effects of HAART can be optimally managed. We recommend the implementation of well controlled cohort studies for the evaluation of long-term effects of HAART treatment on lipid profiles.

## Abbreviations

BMI: Body Mass Index
CI: Confidence Interval
SD: Standard Deviation
TC: Total Cholesterol
TG: Triglycerides
HDL-c: High Density Lipoproteins cholesterol
LDL-c: Low Density Lipoproteins cholesterol
STATA: Statistics/Data Analysis
HAART: Highly Active Antiretroviral Therapy
ART: Antiretroviral Therapy
PLWHA: People Living with HIV/AIDS
3TC: Lamivudine
AZT: Zidovudine
TDF: Tenofovir
NVP: Nevirapine
EFV: Efavirenz
LPV/r: Lopinavir
PI: Protease Inhibitor
NNRTI: Non-Nucleoside Reverse Transcriptase Inhibitor
NRTI: Nucleoside Reverse Transcriptase Inhibitor
CD4^+^: Cluster of Differentiation
